# Thromboxane promotes smooth muscle phenotype commitment but not remodeling of hypoxic neonatal pulmonary artery

**DOI:** 10.1186/s13069-015-0037-6

**Published:** 2015-11-01

**Authors:** Fabiana Postolow, Jena Fediuk, Nora Nolette, Martha Hinton, Shyamala Dakshinamurti

**Affiliations:** Department of Pediatrics, University of Manitoba, 715 McDermot Avenue, Winnipeg, MB R3E 3P4 Canada; Department of Physiology, University of Manitoba, 715 McDermot Avenue, Winnipeg, MB R3E 3P4 Canada; Biology of Breathing Group, Manitoba Institute of Child Health, 715 McDermot Avenue, Winnipeg, MB R3E 3P4 Canada; Section of Neonatology, WS012 Women’s Hospital, 735 Notre Dame Ave, Winnipeg, MB R3E 0L8 Canada

## Abstract

**Background:**

Persistent pulmonary hypertension of the newborn (PPHN) is characterized by vasoconstriction and pulmonary vascular remodeling. Remodeling is believed to be a response to physical or chemical stimuli including pro-mitotic inflammatory mediators such as thromboxane. Our objective was to examine the effects of hypoxia and thromboxane signaling ex vivo and in vitro on phenotype commitment, cell cycle entry, and proliferation of PPHN and control neonatal pulmonary artery (PA) myocytes in tissue culture.

**Methods:**

To examine concurrent effects of hypoxia and thromboxane on myocyte growth, serum-fed first-passage newborn porcine PA myocytes were randomized into normoxic (21 % O_2_) or hypoxic (10 % O_2_) culture for 3 days, with daily addition of thromboxane mimetic U46619 (10^−9^ to 10^−5^ M) or diluent. Cell survival was detected by MTT assay. To determine the effect of chronic thromboxane exposure (versus whole serum) on activation of arterial remodeling, PPHN was induced in newborn piglets by a 3-day hypoxic exposure (FiO_2_ 0.10); controls were 3 day-old normoxic and day 0 piglets. Third-generation PA were segmented and cultured for 3 days in physiologic buffer, Ham’s F-12 media (in the presence or absence of 10 % fetal calf serum), or media with 10^−6^ M U46619. DNA synthesis was measured by ^3^H-thymidine uptake, protein synthesis by ^3^H-leucine uptake, and proliferation by immunostaining for Ki67. Cell cycle entry was studied by laser scanning cytometry of nuclei in arterial tunica media after propidium iodide staining. Phenotype commitment was determined by immunostaining tunica media for myosin heavy chain and desmin, quantified by laser scanning cytometry.

**Results:**

Contractile and synthetic myocyte subpopulations had differing responses to thromboxane challenge. U46619 decreased proliferation of synthetic and contractile myocytes. PPHN arteries exhibited decreased protein synthesis under all culture conditions. Serum-supplemented PA treated with U46619 had decreased G1/G0 phase myocytes and an increase in S and G2/M. When serum-deprived, PPHN PA incubated with U46619 showed arrested cell cycle entry (increased G0/G1, decreased S and G2/M) and increased abundance of contractile phenotype markers.

**Conclusions:**

We conclude that thromboxane does not initiate phenotypic dedifferentiation and proliferative activation in PPHN PA. Exposure to thromboxane triggers cell cycle exit and myocyte commitment to contractile phenotype.

## Background

Interruption of the normal course of postnatal pulmonary circuit relaxation can trigger the respiratory failure and hypoxemia syndrome known as persistent pulmonary hypertension of the newborn (PPHN) [1]. PPHN is characterized by vasoconstriction followed by pulmonary arterial fibrosis [2]. The pathophysiology of PPHN involves multiple pathways of injury, including altered circulating agonist balance, endothelial dysfunction, smooth muscle dysfunction, and phenotype changes [3]. Despite improvements in perinatal care, inflammation due to direct lung injury or sepsis continues to contribute significantly to the disease burden of PPHN. Vasoconstriction is predominant early in the course of PPHN, but arterial structural remodeling and fibrosis progressively and irreversibly increases pulmonary vascular resistance [4]. This remodeling is initiated within days of disease onset in response to a wide variety of stimuli, physical (mechanical stretch or strain) or chemical (hypoxia, vasoactive growth factors). That a third of PPHN patients have partial or unsustained responses to nitric oxide vasodilation [5] suggests rapid onset of remodeling or loss of contractile phenotype. Patients with fixed anatomic narrowing of the pulmonary circulation constitute the majority of non-responders in PPHN therapeutic trials [6].

Cyclooxygenase pathway metabolites contribute to the early pulmonary hypertensive response [7, 8]. Thromboxane A_2_ is an inflammatory prostanoid that binds to G-protein-coupled sarcolemmal thromboxane prostanoid (TP) receptors, leading to increased intracellular Ca^2+^, sensitization of the contractile apparatus to Ca^2+^, and pulmonary vasoconstriction [9]. Meconium-induced lung injury is acutely associated with inflammation and thromboxane release [5]. Thromboxane also mediates septic pulmonary hypertension in the neonate [8, 10]. Diminished cyclooxygenase-1 and prostacyclin synthase activities cause a shift in production of arachidonic acid metabolites toward an increased thromboxane to prostacyclin ratio, causing development of increased pulmonary arterial tone [11]. Thromboxane underlies the early development of pulmonary arterial constriction in chronic hypoxic pulmonary hypertension in newborns [12] and hyperoxia-induced pulmonary hypertension [13]. The inhibition of thromboxane reduces pulmonary hypertension induced by hypoxia [14]. Cyclooxygenase-2 gene knockout exacerbates pulmonary hypertension in chronic hypoxia; this effect is reversed by TP blockade, which attenuates hypoxia-induced pulmonary vasoconstriction [15]. Hypoxia also sensitizes the dose-response relationship of pulmonary arteries to TP stimulation; exposure to chronic hypoxia markedly increases TP-mediated pulmonary vasoconstriction [16].

An etiologic role for thromboxane in vasodilator-unresponsive PPHN is substantiated by clinical data. Increased serum thromboxane is reported in primary and secondary pulmonary hypertensions, and may  correlate with disease severity. An imbalance between thromboxane and prostacyclin production is implicated in pulmonary hypertension accompanying hypoxic respiratory failure, and increases pulmonary vascular resistance [17]. Infants with a primary diagnosis of meconium aspiration have a strong correlation between high pulmonary arterial pressures and serial plasma thromboxane metabolite levels [18, 19]. Infants with PPHN and hypoxemic respiratory failure have higher serum thromboxane to prostaglandin E_2_ ratios, compared with normals [20]. In neonates with hypoxic PPHN treated with extracorporeal membrane oxygenation (ECMO), thromboxane metabolites are elevated in severe cases but decrease concurrently with pulmonary artery pressure upon successful medical management, reflecting progressive resolution of lung injury [21].

A rise in thromboxane is thus associated with worsening PPHN and falling thromboxane with its alleviation. While these data strongly implicate thromboxane as a driver of disease severity, the specific role of thromboxane in arterial remodeling has been difficult to discern. The pulmonary arterial histology of PPHN features muscular layer thickening in small pulmonary arteries and distal extension of muscle to nonmuscularized arteries [22], involving myocyte hypertrophy, hyperplasia, and increased deposition of collagen and elastin [23]. It is arguable that signaling pathways for pulmonary vasoconstriction and proliferative activation, including those mediated by inflammation and thromboxane, have significant overlap [24].

In this study, to examine the effect of thromboxane on the initiation of PPHN arterial remodeling, we combined in vitro thromboxane challenge of hypoxic pulmonary arterial smooth muscle with organ culture studies on pulmonary vessels from PPHN and control animals. This approach has been used elsewhere to study vascular cell differentiation and signaling, while maintaining cell contact and cell-matrix interactions in an intact three-dimensional tissue environment [25, 26]. We hypothesized that thromboxane promotes cell cycle entry of synthetic phenotype myocytes in the pulmonary arterial wall and that this effect would be pronounced under hypoxic conditions.

## Methods

### PPHN model

All animal protocols were cleared by the University of Manitoba Committee on Animal Use, within the guidelines of the Canadian Council on Animal Care. To induce hypoxic PPHN, newborn piglets from a pathogen-free farm supplier (<24 h of life; *n* = 5) were housed in normobaric hypoxic (FiO_2_ 0.10, by admixture of room air with N_2_) for 72 h in a sealed thermoregulated isolette with appropriate diurnal cycling. The chamber was opened for no more than 1 h a day for feeding and cleaning. The use of half-atmospheric O_2_ tension is well-established to induce pulmonary hypertension [27], and is extensively studied in our hands [28–30]. Controls included age-matched normoxic piglets obtained at 3 days of age (*n* = 5) and newborn piglets (<24 h, *n* = 5). At the conclusion of environmental exposure, piglets were killed by pentobarbital overdose and exsanguination. Heart and lungs were removed en bloc and placed in oxygenated cold (4 °C) Ca^2+^-free Krebs-Henseleit physiological buffer containing (in mM) the following: 112.6 NaCl, 25 NaHCO_3_, 1.38 NaH_2_PO_4_, 4.7 KCl, 2.46 MgSO_4_∙7H_2_O, and 5.56 dextrose (pH 7.4).

### Primary pulmonary artery myocyte culture

Newborn piglets (<24 h age, *n* = 4) were euthanized as above for primary culture of pulmonary arterial myocytes. Second- to sixth-generation pulmonary arteries were obtained by microdissection into Ca^2+^-free Krebs-Henseleit physiological buffer. Arteries were permitted to recover in cold Hepes buffered saline (HBS) solution (in mM: 130 NaCl, 5 KCl, 1.2 MgCl_2_, 10 HEPES, and 10 glucose; pH 7.4) supplemented with an antibiotic-antimycotic mixture and gentamicin then washed with Ca^2+^-reduced HBS (20 μM CaCl_2_) and finely minced. Tissue was transferred to digestion medium containing Ca^+2^-reduced HBS, type I collagenase (1750 U/ml), dithiothreitol (1 mM), bovine serum albumin (BSA, 2 mg/ml), and papain (9.5 U/ml) for 15 min at 37 °C with gentle agitation. Dispersed pulmonary arterial smooth muscle cells were collected by centrifugation at 1200 rpm for 5 min, washed in Ca^+2^-free HBS, and resuspended in Ham’s F-12 medium supplemented with l-glutamine, 10 % fetal calf serum, 1 % streptomycin, and 1 % penicillin. Cells were seeded on 100-mm plates at density 1 × 10^5^ cells/mm and utilized at first passage.

### Cell survival

Primary myocytes were passaged onto 96-well plates then serum-deprived for 2 days (in Ham’s F-12 medium with l-glutamate-penicillin-streptomycin and 1 % insulin-transferrin-selenium) to synchronize in a contractile phenotype or grown in serum-supplemented media (10 % fetal calf serum) to maintain a synthetic phenotype. Serum-starved or serum-supplemented plates were then randomized to growth in normoxia (21 % O_2_, 5 %CO_2_) or hypoxia (10 % O_2_, 5 % CO_2_) for 72 h. During this time, cells were exposed to U46619 (thromboxane mimetic) at graded concentrations (10^−9^–10^−5^ M) or diluent, in the presence or absence of 10^−6^ M thromboxane antagonist SQ29548. Smooth muscle monoculture was confirmed by immunostaining for α-sm-actin; differentiation into contractile phenotype was confirmed by immunostaining for smooth muscle myosin heavy chain. Cell survival was determined by MTT Assay Kit (Cayman Chemical). Briefly, cells were incubated with 20 μl MTT reagent in 100 μl culture media for 3 h at 37 °C, followed by reaction termination. The bioreduced reaction product was quantified at 570 nm using a microplate reader. To identify any off-target effects of U46619 stimulation on prostaglandin reuptake, PGE2 concentration was determined in conditioned media of serum-deprived normoxic and hypoxic myocytes incubated for 4 h with 10^−6^ M U46619 or buffer, in the presence or absence of 10^−6^ M SQ29548 by ELISA (Life Technologies).

### ^3^H-thymidine incorporation

First-passage serum-starved or serum-supplemented myocytes on 12-well plates as described above were randomized to growth in normoxia (21 % O_2_, 5 %CO_2_) or hypoxia (10 % O2, 5 % CO_2_) for 72 h, in the presence or absence of 10^−6^ M U46619. Culture media was then removed and 1 μCi of ^3^H-thymidine (thymidine-[methyl-^3^H] specific activity 6.7 Ci/mmol; unit size 250 μCi; Perkin Elmer) in 1 ml fresh media was added to each well for 4 h of incubation in room air (21 % O_2_). Cells were washed several times with 9 % NaCl then fixed with 1 ml ice-cold 5 % trichloroacetic acid for 30 min. The resulting precipitate was solubilized in 1 M NaOH at room temperature. Solubilized DNA was transferred into scintillation vials, and ^3^H-thymidine uptake was quantified by scintillation counting, expressed as dpm/10^5^ cells.

### Ex vivo PA tissue culture

Strips (1 cm) of third intrapulmonary generation neonatal pulmonary arteries from PPHN and control piglets (9–12 strips per animal) were gently dissected under sterile conditions, denuded of endothelium and cut longitudinally into halves. The strips were randomized to be placed in 1 ml of sterile physiologic buffer (Ca^2+^-free Krebs-Henseleit), serum-supplemented culture medium (Ham’s F-12 supplemented with l-glutamate, 10 % fetal calf serum, 1 % streptomycin, and 1 % penicillin), or serum-deprived culture medium (Ham’s F-12 with l-glutamate-penicillin-streptomycin, and 1 % insulin-transferrin-selenium) for 72 h. All organ cultures were incubated at 37 °C in normoxic conditions; during this time, half of the strips also received treatment with 10^−6^ M U46619.

### DNA and protein synthesis

At the conclusion of the 3-day incubation as above, pulmonary artery organ cultures were exposed for 24 h to ^3^H-thymidine (thymidine-[methyl-^3^H] specific activity 6.7 Ci/mmol; unit size 250 μCi; Perkin Elmer) or ^3^H leucine (l-leucine [4,5-^3^H (N)], specific activity 40–60 Ci/mmol; unit size 250 μCi; Perkin Elmer) at activities 1 μCi/ml, respectively, in fresh media or physiologic buffer. Tissues were liberally washed with 9 % NaCl and digested in 200 μl 2 M NaOH. Protein concentrations of tissue homogenates were determined by Bradford method, using bovine serum albumin as a standard. Scintillation fluid was added to 100 μl tissue homogenate and radioactivity was measured in a liquid scintillation counter, expressed as cpm/mg of protein.

### Immunofluorescence studies

Following organ culture as described above for 72 h in media with or without serum, and in the presence or absence of stable thromboxane mimetic U46619, pulmonary artery segments were fixed in 4 % formaldehyde, washed in PBS, embedded in OCT Compound (Tissue-Tek), frozen slowly to prevent tissue fracture, and stored at −80 °C until use. Sections were cut at 10-μm thickness on a cryostat, air-dried and kept at −20 °C, and then permeabilized in 0.5 % Triton X-100 for 15 min at room temperature. Nonspecific antibody binding was blocked with 10 % donkey serum in cyto-TBS (in nM: 20 Tris base, 154 NaCl, 2 EGTA, 2 MgCl2, pH 7.4) for 30 min at room temperature in a humidified chamber. Sections were evaluated for Ki67 (nuclear marker of proliferating cells) by immunostaining for Ki67 (diluted 1:50; BD Pharmingen) and α-actin (1:100; Sigma) followed by incubation with FITC-conjugated donkey anti-rabbit antibody and Cy3-conjugated donkey anti-mouse antibody. Slides were evaluated by epifluorescence microscopy by an observer blinded to treatment group; results were expressed as Ki67 positive nuclei/mm^2^. For evaluation of myocyte contractile machinery, sections were incubated overnight at 4 °C with anti-myosin heavy chain (1:100; Sigma) and anti-desmin (1:100; Sigma). For examination of apoptosis, sections were incubated with anti-cleaved caspase-3 (1:200; Sigma) and anti-BAX (1:50; Santa Cruz Biotechnology), followed by the same secondary antibodies. Sections were mounted with antifade and staining intensity quantified by laser scanning cytometry (LSC; iCYS ™ Research Imaging Cytometer), using Olympus IX-71 microscope at ×40 objective. Contouring and event segmentation were adjusted for optimal fluorescence detection, at standardized fluorescence integral and maximum pixel fluorescence, in six to eight equally sized regions of interest per slide with minimal cell-free areas. Results were expressed as integral intensity/mm^2^.

### Cell cycle entry

Sections were briefly hydrated in 2× sodium chloride citrate buffer (2× SSC; 0.3 M NaCl, 0.03 M Na citrate; pH 7.0) and then incubated in 100 μg/ml DNase-free RNAse in 2× SSC (ribonuclease A; Sigma) for 20 min at 37 °C. Following several rinses, tissues were incubated with propidium iodide (PI; 1:3000 in 2× SSC; Molecular Probes) at room temperature in the dark for 5 min. Sections were then mounted with antifade and visualized by LSC at ×40 magnification, 488 nm excitation, and 565–595 nm emission to capture PI staining of nuclear DNA [31, 32]. Gating was adjusted and standardized for optimal detection of fluorescence events; data expressed as integral fluorescence/event/cell count was calculated as percentage of cells in 2N and 4N DNA cell cycle phases. Pyknotic nuclei were quantified from the scattergram as highest intensity (max pixel) events.

### Statistical analysis

Statistical analyses utilized ANOVA with post-test Bonferroni correction for multiple comparisons; *p* < 0.05 was considered statistically significant. Data was presented as mean ± SEM.

## Results

### Effect of thromboxane exposure on pulmonary artery myocyte survival and growth

**Fig. 1:**
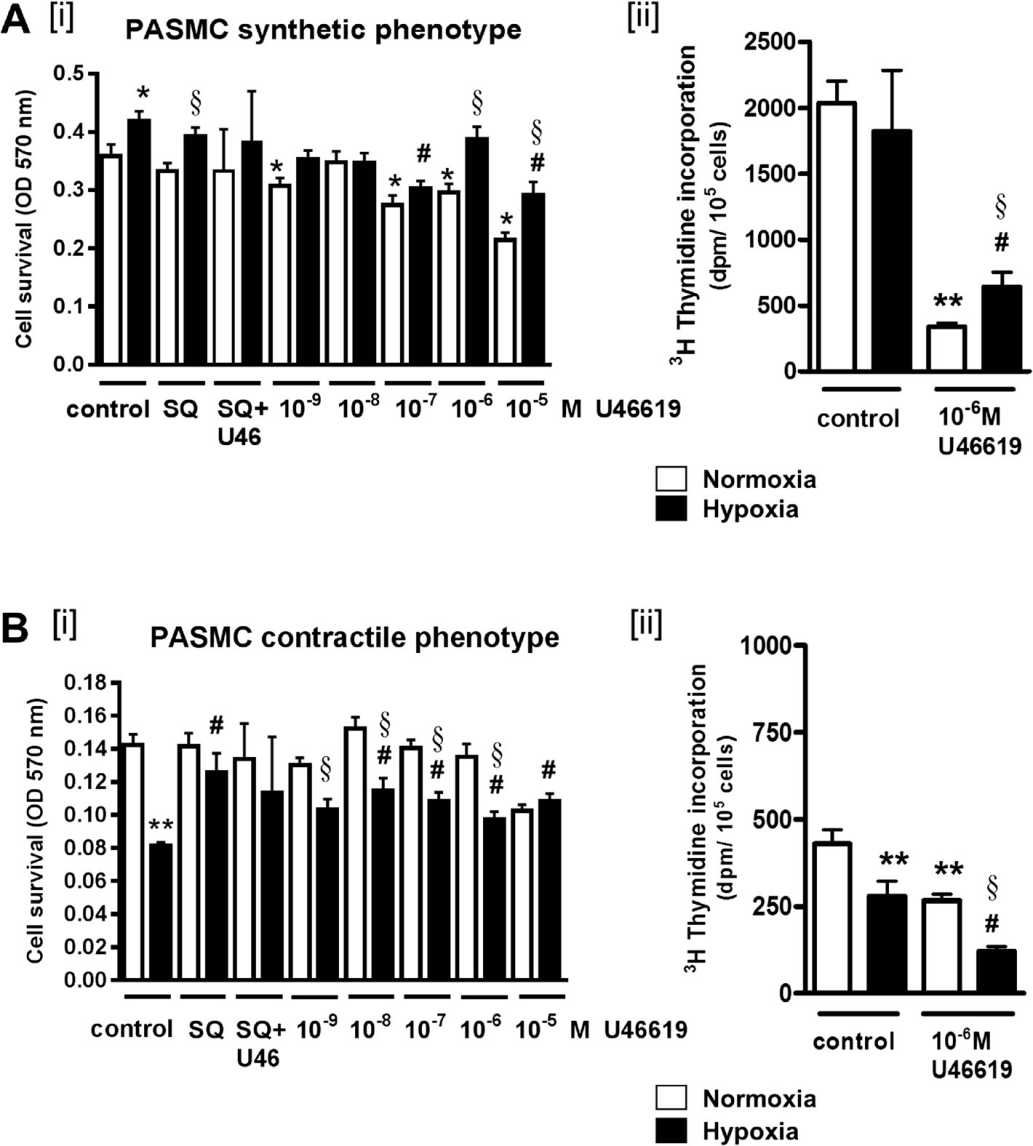
First-passage pulmonary artery myocytes on 96-well plates, allowed to adhere for 24 h, then serum-starved for 2 days to synchronize in a contractile phenotype, or maintained serum-fed (synthetic phenotype), followed by normoxic (21 % O_2_, 5 % CO_2_) or hypoxic (10 % O_2_, 5 % CO_2_) culture conditions for 72 h, with serial concentrations (10^−9^ to 10^−5^ M) of the stable thromboxane mimetic U46619, or 10^−6^ M SQ29548 (SQ; thromboxane receptor antagonist) added daily for 3 days. Cell survival by MTT assay; single-point analysis of DNA synthesis by ^3^H-thymidine uptake. **a** *[i]* Cell survival in synthetic phenotype normoxic and hypoxic myocytes; growth in the presence of SQ, SQ plus 10^−5^ M U46619, or serial concentrations of U46619. *[ii]* ^3^H-thymidine incorporation in normoxic and hypoxic synthetic myocytes following exposure to U46619. **b** *[i]* Cell survival in contractile phenotype myocytes during hypoxia, in SQ, SQ plus 10^−5^ M U46619, or increasing concentrations of U46619. *[ii]* ^3^H-thymidine incorporation in normoxic and hypoxic contractile myocytes during hypoxia and concomitant U46619 (in all panels, *p < 0.01 and **p < 0.001 compared to normoxic untreated control, #*p* < 0.01 compared to hypoxic untreated; §*p* < 0.05 compared to normoxic cells in same treatment group)

**Fig. 2:**
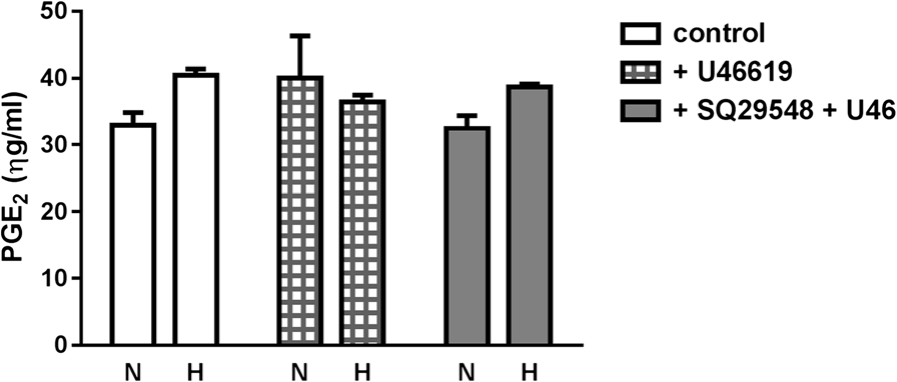
First-passage pulmonary arterial myocytes grown in normoxia or hypoxia for 3 days (without change of media), and incubated with 10^−6^ M U46619 or diluent, with or without 10^−6^ M TP antagonist SQ29548 for 4 hours. PGE2 measured in conditioned media by ELISA (*p* = ns)

The effects of hypoxia and U46619 on cell survival were evaluated by MTT assay. First-passage pulmonary artery myocytes were either serum-deprived for 2 days to synchronize in a contractile phenotype or maintained serum-fed (synthetic phenotype). Then, all groups were randomized to normoxic (21 % O_2_, 5 % CO_2_) or hypoxic (10 % O_2_, 5 % CO_2_) culture conditions for 72 h and simultaneously exposed to graded concentrations (10^−9^ to 10^−5^ M) U46619 added daily for 3 days. Cell survival was quantified by MTT colorimetric assay. All plates studied were near 80 % confluence. Smooth muscle phenotype was routinely confirmed by immunostaining for smooth muscle α-actin and myosin heavy chain (not shown; similar phenotyping data published [30]). Cell survival was greater in serum-fed (synthetic) hypoxic myocytes than in normoxic myocytes; this effect was significant upon growth in high concentrations of U46619 (Fig. 1a(i)). Route of action of high dose U46619 was verified by sensitivity to TP blockade. In contractile phenotype myocytes, cell survival was diminished during hypoxia and after incubation in U46619 (Fig. 1b(i)). For a single-point analysis of DNA synthesis, cells were incubated with 1 mCu per well ^3^H-thymidine in fresh media added for 4 h at the end of environmental exposures. ^3^H-thymidine uptake (as dpm/10^5^ cells) was comparable in normoxic and hypoxic synthetic phenotype myocytes. U46619 exposure decreased thymidine incorporation in normoxic and hypoxic synthetic myocytes, to a level consistent with that of contractile myocytes; suppression of DNA synthesis was greater in normoxic cells (Fig. 1a(ii)). Contractile myocytes exhibited low thymidine incorporation compared to the synthetic myocytes, and this decreased further during hypoxia and concomitant U46619 (Fig. 1b(ii)). Off-target effects of U46619 on prostacyclin transporter-mediated PGE2 reuptake were determined in conditioned media (Fig. 2). Neither U46619 nor concurrent TP blockade had any effect on media PGE2 accumulation. PGE2 concentration was also not altered by hypoxia.

### Effect of thromboxane exposure during PPHN or control pulmonary artery tissue culture on cell growth and protein synthesis

**Fig. 3:**
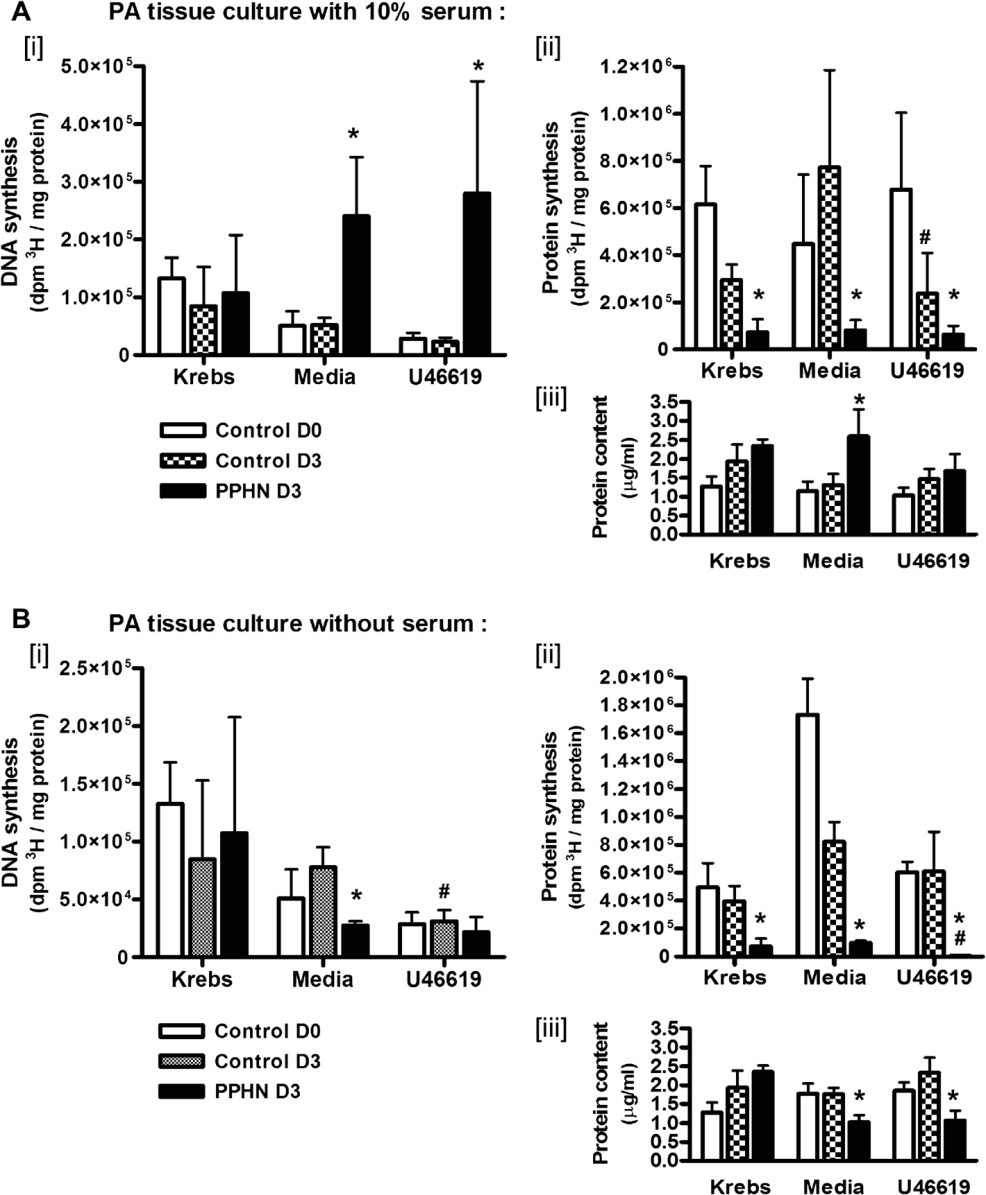
Third-generation pulmonary artery strips from newborn (control D0), normoxic 3-day-old (control D3), and hypoxic 3-day-old piglets (PPHN D3) cultured for 72 h in aerated media with or without 10 % fetal bovine serum (favoring expansion of synthetic or contractile phenotype, respectively). Tissue culture in Krebs physiological buffer also studied as a baseline control. Tissue strips were randomized to daily addition of thromboxane mimetic U46619 for 72 h. Tissue DNA synthesis was evaluated by ^3^H-thymidine incorporation and protein synthesis by incorporation of ^3^H-leucine. Labeled amino acid or nucleotide was added for the final 24 h; tissue uptake expressed is as dpm/mg protein. Protein contents for tissue homogenates from all groups were obtained by Bradford method. **a** *[i]* DNA synthesis, *[ii]* active protein synthesis in PPHN PA cultured in media with serum, and after chronic exposure to U46619. **b** In serum-deprived tissues, decreased DNA synthesis *[i]* and active protein synthesis *[ii]* especially following U46619 exposure. Protein content was lower in homogenates of PPHN arteries in media and in U46619 (*N* = 5; *p < 0.05 compared to control D3 in same treatment group; #*p* < 0.05 compared to PA from same animal model in media alone)

Third-generation intrapulmonary arteries from newborn (day 0), 3-day-old control or 3-day hypoxic (PPHN) piglets were maintained in either aerated Krebs buffer, culture medium with 10 % fetal bovine serum, or culture medium without serum, and randomized to a daily treatment with 10^−6^ M U46619 or diluent for 3 days. To evaluate DNA synthesis, ^3^H-thymidine was added to culture media in the final 24 h; to quantify protein synthesis, ^3^H-leucine was added to media for 24 h. DNA synthesis increased in PPHN arteries compared to age-matched control arteries when cultured in media with serum and after chronic exposure to U46619 (*p < 0.05; Fig. 3a(i)). In serum-starved tissues, DNA synthesis and active protein synthesis were low under all conditions when compared to serum-fed tissues, and further attenuated by U46619 exposure (Fig. 3b(i), b(ii)). Active protein synthesis was decreased in PPHN arteries compared to 3-day-old controls in all tissue culture conditions (*p < 0.05; Fig. 3a(ii), b(ii)).

### Effect of thromboxane exposure on cell cycle entry in serum-fed PPHN or control pulmonary artery

**Fig. 4:**
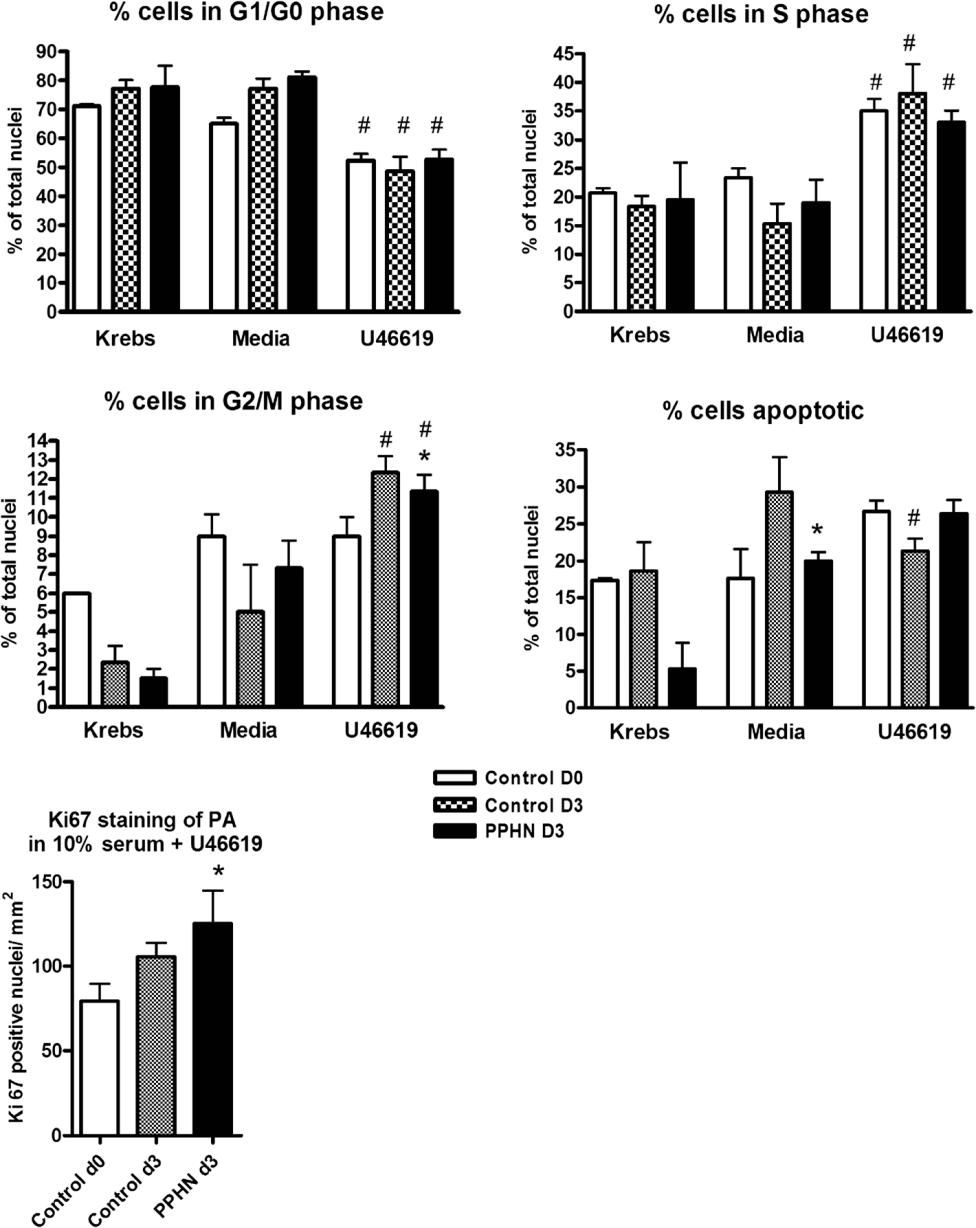
Third-generation pulmonary artery strips from newborn (control D0), normoxic 3-day-old (control D3), and hypoxic 3-day-old piglets (PPHN D3) cultured for 72 h in aerated media with 10 % fetal bovine serum (favoring synthetic phenotype) with daily addition of thromboxane mimetic U46619 or diluent for 72 h. Tissues also studied in Krebs physiological buffer as a baseline control. Nuclei stained with propidium iodide. Proportion of nuclei in each cell cycle stage (quantified by laser scanning cytometry) was calculated as percent cells in 2N and 4N DNA phases and expressed as integral fluorescence/event/cell count. Apoptosis quantified as percent pyknotic nuclei (low area high fluorescence). Mitotic nuclei in medial layer of control and PPHN arteries quantified after exposure to U46619, as Ki67-positive nuclei/mm^2^ (in all panels, *p < 0.05 compared to control D3 in same treatment group; #*p* < 0.05 compared to PA from same animal group in media alone)

The presence of U46619 in serum-fed tissues during normoxia or hypoxia decreased the percentage of cells in G1/G0 stage from approximately 80 to 55 % (*p < 0.01). Cells in S stage increased in the presence of U46619 compared to media controls (*p < 0.01). Cells in G2/M cells were of relatively low frequency, lowest in tissues maintained in physiological buffer, increased in media, and significantly increased after U46619 stimulation (Fig. 4). This was substantiated by increased Ki67-positive nuclei in PPHN arteries chronically exposed to U46619, compared to age-matched control arteries exposed to U46619.

### Effect of thromboxane exposure on cell cycle entry in serum-starved PPHN or control pulmonary artery

**Fig. 5:**
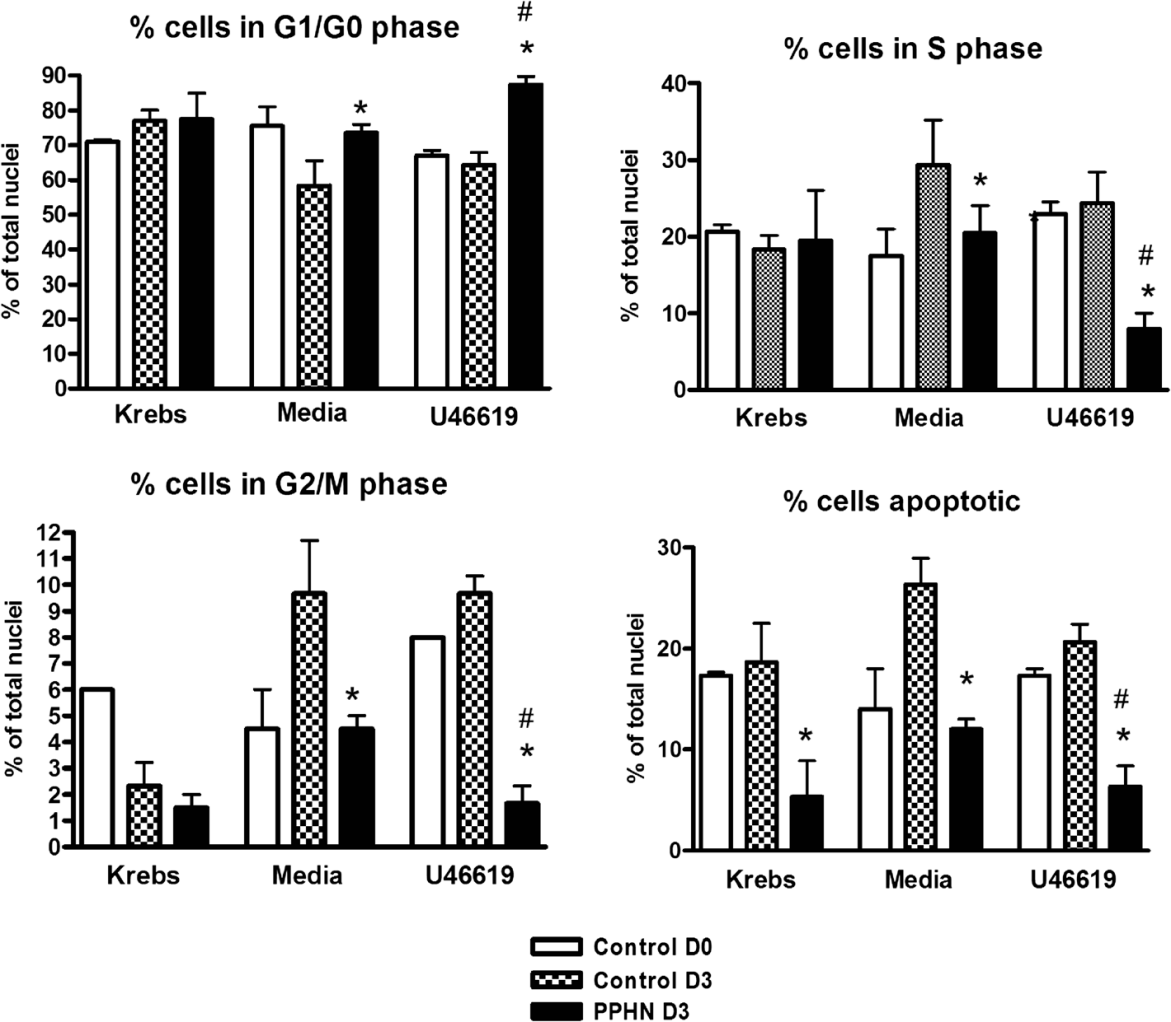
Third-generation pulmonary artery strips from newborn (control D0), normoxic 3-day-old (control D3), and hypoxic 3-day-old piglets (PPHN D3) cultured for 72 h in aerated media without serum (synchronizing cells in contractile phenotype) with daily addition of thromboxane mimetic U46619 or diluent for 72 h. Tissues studied in Krebs physiological buffer served as a baseline control. Nuclei were stained with propidium iodide. Proportion of nuclei in each cell cycle stage (quantified by laser scanning cytometry) was calculated as percent cells in 2N and 4N DNA phases and expressed as integral fluorescence/event/cell count. Apoptosis was quantified as percent pyknotic nuclei (low area, high intensity). Mitotic nuclei in medial layer of control and PPHN arteries quantified after exposure to U46619, as Ki67-positive nuclei/ mm^2^ (in all panels, *p < 0.05 compared to control D3 in same treatment group; #*p* < 0.05 compared to PA from same animal group in media alone)

The percentage of G1/G0 stage cells in the media of arteries cultured in buffer or in serum-free media was similar. PPHN arteries had fewer cells in G2/M phase, and when chronically exposed to U46619, they also had fewer cells in S phase and an increased percentage of cells in G0/G1 (Fig. 5). Minimal Ki-67 staining was seen in serum-deprived tissues (data not shown).

### Effect of thromboxane exposure on apoptosis in PPHN or control pulmonary artery

**Fig. 6:**
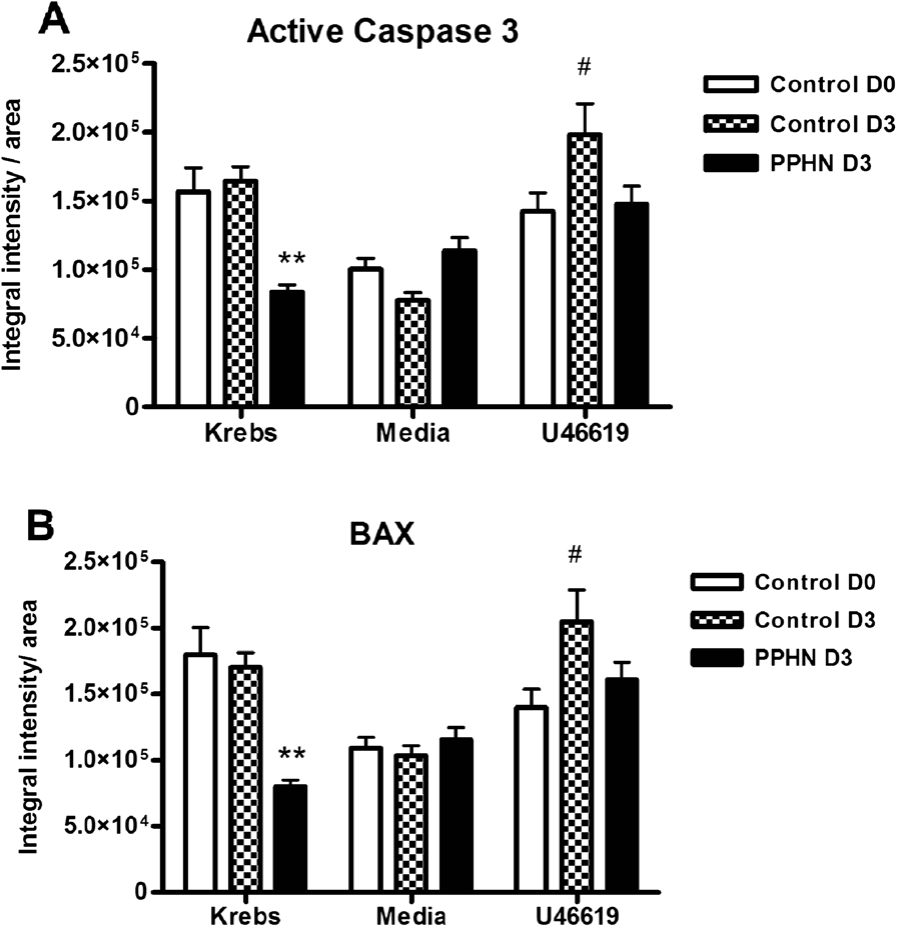
Immunostaining for apoptotic markers, active caspase 3 (**a**) and BAX (**b**), was analyzed by laser scanning cytometry in frozen sections of pulmonary artery from control and PPHN animals, following chronic exposure to U46619 added daily for 72 h in the absence of serum. Results expressed as integral intensity/mm^2^ area (**p < 0.001 compared to control D3 in same treatment group; #*p* < 0.01 compared to PA from same animal group in media alone)

Immunostaining for activated caspase 3, a final effector of apoptosis (Fig. 6a), and BAX, a key pro-apoptotic protein (Fig. 6b), was analyzed by laser scanning cytometry in cultured PPHN and control pulmonary arteries chronically exposed to U46619 in the absence of serum supplementation. Tissues cultured in media had similar levels of apoptosis markers. Activation of caspase-3 and BAX abundance increased in control pulmonary arteries exposed to U46619, but this did not occur in PPHN arteries.

### Effect of thromboxane exposure on phenotype of PPHN or control pulmonary artery

**Fig. 7:**
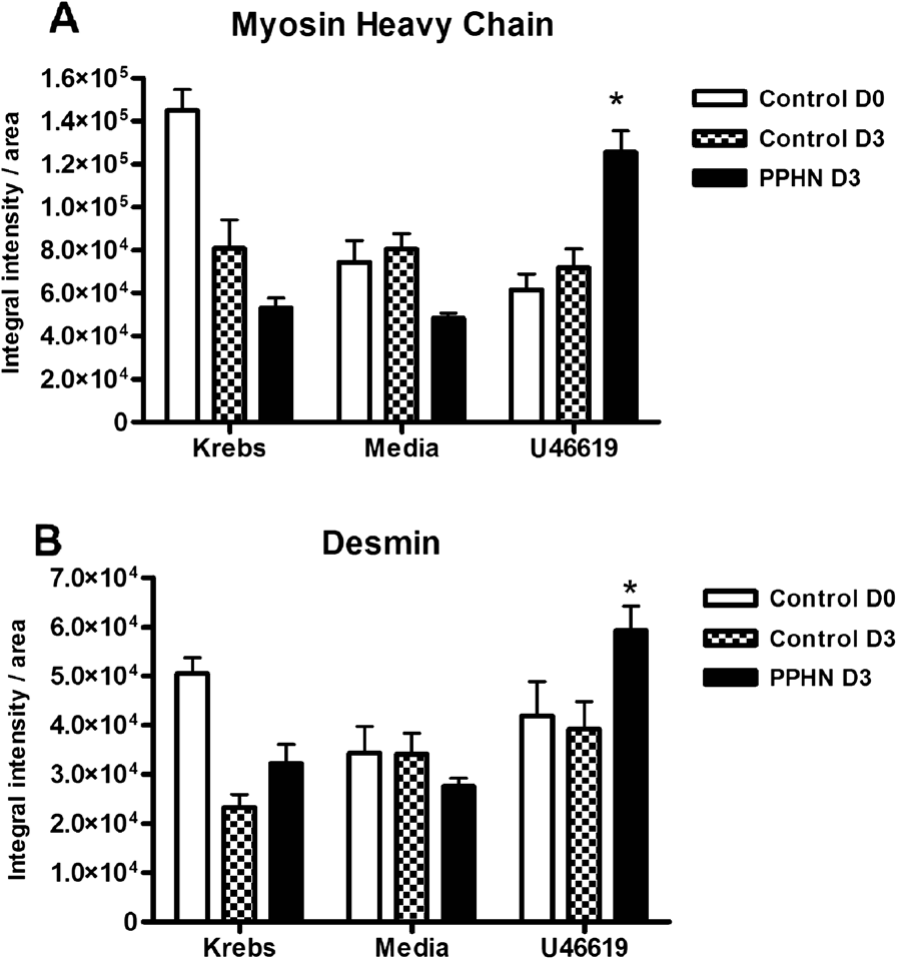
Immunostaining for contractile protein markers, myosin heavy chain (**a**) and desmin (**b**), was analyzed by laser scanning cytometry in frozen sections of pulmonary artery from control and PPHN animals, following chronic exposure to U46619 added daily for 72 h in tissue culture in the absence of serum. Results expressed as integral intensity/mm^2^ area (*p < 0.01 compared to age-matched control)

Immunostaining for contractile phenotype marker myosin heavy chain (Fig. 7a) and desmin (Fig. 7b) was quantified in PPHN and control pulmonary arteries by laser scanning cytometry of frozen sections following tissue culture in the absence of serum supplementation and after chronic exposure to U46619. Myosin heavy chain and desmin staining was slightly decreased in PPHN arteries compared to controls when cultured in media alone; but in the presence of U46619, staining for these contractile phenotype markers increased dramatically.

## Discussion

In this study, we examined smooth muscle proliferative responses engendered by hypoxia and thromboxane, two potent drivers of neonatal PPHN, in a piglet model of neonatal pulmonary hypertension. Based on in vitro studies and ex vivo tissue culture of neonatal pulmonary arteries, we conclude that contractile and synthetic phenotype smooth muscle subpopulations in PPHN had differing responses to a thromboxane challenge. Pulmonary artery myocytes studied in a synthetic phenotype had slightly increased survival and proliferation during hypoxia, but the presence of U46619 decreased proliferation of synthetic myocytes to lower levels seen in contractile myocytes. Contractile phenotype pulmonary myocytes grown in hypoxia had slightly decreased survival and proliferation compared to those in normoxia, with further attenuation upon incubation with U46619. Tissue culture indicated that PPHN arteries exhibited decreased protein synthesis under all culture conditions (DNA synthesis increased in serum-supplemented PPHN pulmonary arteries exposed to U46619 but decreased in serum-deprived PPHN arteries). In serum-supplemented arteries, independent of disease phenotype, the percentage of cells in G1/G0 phase decreased during tissue culture in the presence of U46619, with an increase in cells in S and G2/M phase. Among serum-deprived arteries, PPHN arteries exhibited arrest of cell cycle progression following U46619 challenge, with an increase in G0/G1 and a decrease in S and G2/M phases. PPHN arteries incubated with U46619 also showed evidence of increased contractile phenotype commitment of smooth muscle.

We interpret these findings to mean that thromboxane does not initiate the phenotypic dedifferentiation and proliferative activation that are the hallmark of pulmonary arterial fibrosis in PPHN; on the contrary, exposure to thromboxane triggers cell cycle exit and possibly expansion of the contractile smooth muscle phenotype population. This is particularly evident during arterial organ culture in the absence of serum, which replicates the normal in vivo environment of the endothelium-protected arterial media.

It has long been understood that inflammatory mediators stimulate pathways favoring both muscle contraction and proliferation. In addition to its vasoconstrictor role, thromboxane is known to have mitogenic effects [33]. Increased endothelial production of thromboxane is associated with vasoconstriction as well as with smooth muscle hypertrophy leading to vascular remodeling. Loss of endothelial integrity also results in contact of serum proliferative mediators with underlying vascular tissue, causing medial and adventitial hypertrophy [34]. Hypoxia may disturb endothelial function by altering regulation of vascular tone, increasing permeability and promoting release of growth factors [35]. In this study, we isolated the role of thromboxane by adding it to a monotypic smooth muscle culture and to an organ culture of endothelium-denuded pulmonary artery, thereby excluding other endothelial growth factors. In this model, thromboxane did not prove to be mitogenic, as it sustained smooth muscle contractile phenotype commitment without promoting dedifferentiation or proliferation. We also sought to identify indirect action of U46619 on prostaglandin transporter activity, as a U46619-mediated increase in media PGE2 or PGI2 could theoretically influence any observed anti-proliferative effects [36]. We observed no accumulation of media PGE2 following the 72-h hypoxia or U46619 challenge; we have previously reported the same for PGI2 [37]. Others have also reported insensitivity of PGE2 reuptake to U46619 [38]. We therefore exclude this off-target action and focus on the effect of TP activation.

Vascular remodeling denotes morphological changes of the vessel wall in response to noxious stimuli inducing reorganization of the vessel wall structure, luminal narrowing, and circuit hypoperfusion. Clinical triggers for adaptive remodeling include hemodynamic stress, mechanical injury, inflammation, and hypoxia [39]. In later pulmonary hypertension, the poor response to vasodilators may represent relative loss of the contractile component, as the increase in vascular resistance is due to mural fibrosis, structural narrowing, and obliteration of small distal pulmonary arteries [40]. As the major component of vascular media, smooth muscle is the main effector of pulmonary arterial remodeling [41]. Subpopulations of pulmonary arterial myocytes with differing proliferative potentials in response to injury are distinguishable early in life [42]. These populations are distinct with respect to their state of differentiation, expression of smooth muscle markers, and proliferative responses to growth factors and hypoxia [43]. Based on functional physiology, morphology, and immunohistochemistry, at least four smooth muscle phenotypes are reported in the pulmonary arterial media [4]. The extremes of the phenotype continuum are the fully contractile muscle cell with few synthetic organelles and no capability of proliferation, and the synthetic smooth muscle phenotype that is migratory, secretory, and capable of division but with little contractile machinery [44]. Under non-disease conditions, the majority of myocytes exist in a contractile phenotype but quiescent state, and only vascular injury causing exposure to serum factors activates the altered gene expression necessary for cumulative hypertrophy or hyperplasia [45]. Medial myocytes can be activated from cell cycle arrest into a synthetically active and proliferative phenotype [46]. This involves complex regulation of entry into the cell cycle, a series of tightly regulated steps that control DNA synthesis and mitosis. The resting (contractile) myocyte is maintained in a non-proliferative gap phase (G0); the activated myocyte enters the G1 phase where the necessary elements are assembled to permit entry into the synthetic phase (S phase) of DNA replication. Cells subsequently enter a second gap phase (G2) during which proteins that are used in mitosis are synthesized [47]. In our model, exposure of both PPHN and control pulmonary arteries to serum during organ culture induced cell cycle progression, and this was increased in the presence of exogenous thromboxane; this models the effect of loss of endothelial integrity. Ki67 staining of mitotic cells was also increased in serum-fed PPHN arteries. However, upon tissue culture in the absence of serum (which models the undamaged vessel), cell cycle entry was halted in PPHN arteries, DNA synthesis decreased, and protein synthesis diminished relative to age-matched controls. Also in the absence of serum, addition of exogenous thromboxane attenuated cell cycle commitment. Increased immunostaining for contractile protein markers in PPHN vessels following thromboxane stimulation indicates thromboxane did not signal for dedifferentiation of smooth muscle in the medial layer. This goes against literature implicating thromboxane [48] and thromboxane receptor expression [49] in hypoxic pulmonary arterial remodeling and loss of distensibility, and in rapid cell cycle progression of myocytes [50]. However, this finding was supported by data from cultured myocytes pre-synchronized by serum deprivation or supplementation in contractile or synthetic phenotype. DNA synthesis was naturally greater in synthetic myocytes than contractile; but the presence of U46619 virtually halted synthetic myocyte thymidine uptake. Contractile myocytes had low DNA synthesis under hypoxic conditions, with a small further decrease after U46619 incubation. Examining the balance of survival versus apoptosis, hypoxia and U46619 increased synthetic myocyte survival and decreased contractile myocyte survival. PPHN vessels had a decreased abundance of apoptotic markers, and in the presence of serum, greater DNA synthesis (albeit lower protein synthesis) in tissue culture indicating mural myocytes could be induced toward proliferation in the presence of the correct stimuli; however, in the absence of serum, thromboxane alone suppressed DNA synthesis in PPHN vessels. We interpret this to indicate that thromboxane is not the serum element responsible for smooth muscle proliferative signaling. When the majority population of the arterial wall is muscle resting in the contractile phenotype, thromboxane appears to promote cell cycle exit and differentiation, with its attendant decrease in proliferation and DNA synthesis. Further explication can be derived from studies linking thromboxane-mediated mitogenic signaling with required co-stimulation by other growth factors including platelet-derived growth factor [51], oxidized lipoproteins [52], or other vasopressors [53] and prevention of thromboxane-mediated proliferation by polyunsaturated fatty acids [54]. Thromboxane in isolation may act mainly as a vasoconstrictor but may require other concurrent stimulation to act as a mitogen. In addition, two TP receptor affinity subtypes are identified, the higher affinity site associated with thromboxane-induced vasoconstriction and the lower affinity site with smooth muscle proliferation [55]; we speculate that exogenous U46619 could trigger signaling via high-affinity TP for vasoconstriction, without activation of synthetic myocytes.

The novelty of this study lies in the combination of in vitro and ex vivo methods with an analysis of cell cycle entry to determine the specific role of thromboxane (as opposed to associated serum factors) in smooth muscle proliferative signaling. There are limitations inherent in the use of ex vivo organ culture to study the effect of prolonged thromboxane stimulation on pulmonary arterial signaling. However, vascular tissue culture has been used to investigate DNA and protein synthesis in many different vessel types [56–59], maintaining tissue viability, contractility, and relaxation for up to 5 days [59, 60]. Serum deprivation of cultured vessel strips induces maximal force responses, while supplementation with serum promotes tissue growth and DNA synthesis, with decreased capacity for force generation [61]; this manipulation is similar to the effects of in vitro serum deprivation or supplementation of cultured myocytes. Organ culture methods offer greater control of experimental conditions than in vivo studies while maintaining the three-dimensional cell environment and avoiding disruption of tissue integrity [62].

## Conclusions

We conclude inflammation and hypoxia appear to influence myocyte proliferation in a cell type-specific manner. Hypoxia is a potent stimulus of smooth muscle proliferation and pulmonary arterial remodeling. However, the presence of thromboxane increases smooth muscle commitment to a contractile phenotype—this may worsen vasoconstriction in PPHN. Despite our initial hypothesis that elevated serum thromboxane promotes pulmonary vascular fibrosis in PPHN, our data provide new evidence that the primary driver of pulmonary artery medial proliferation is hypoxia and that co-stimulation with thromboxane is not additive. Therapeutic strategies for PPHN targeting vascular remodeling may need to take into account the effects of inflammation.

## Abbreviations

IP: prostacyclin receptor
PGE_1_, PGE_2_: prostaglandin
PGI_2_: prostacyclin
PPHN: persistent pulmonary hypertension of the newborn
SQ29548: [1S-[1α,2α(Z),3α,4α]]-7-[3-[[2-[(phenylamino)carbonyl]hydrazino]methyl]-7-oxabicyclo[2.2.1]hept-2-yl]-5-heptenoic acid; thromboxane receptor antagonist
TP: thromboxane prostanoid
TxA_2_: thromboxane A_2_
U46619: 9,11-dideoxy-9a,11a-methanoepoxy prostaglandin F2α, a thromboxane mimetic
